# Considering the possibilities and pitfalls of Generative Pre-trained Transformer 3 (GPT-3) in healthcare delivery

**DOI:** 10.1038/s41746-021-00464-x

**Published:** 2021-06-03

**Authors:** Diane M. Korngiebel, Sean D. Mooney

**Affiliations:** 1 The Hastings CenterGarrison, New York, NY USA; 2 Department of Biomedical Informatics and Medical Education, University of Washington Seattle, Seattle, WA USA

**Keywords:** Technology, Ethics

## Abstract

Natural language computer applications are becoming increasingly sophisticated and, with the recent release of Generative Pre-trained Transformer 3, they could be deployed in healthcare-related contexts that have historically comprised human-to-human interaction. However, for GPT-3 and similar applications to be considered for use in health-related contexts, possibilities and pitfalls need thoughtful exploration. In this article, we briefly introduce some opportunities and cautions that would accompany advanced Natural Language Processing applications deployed in eHealth.

A seemingly sophisticated artificial intelligence, OpenAI’s Generative Pre-trained Transformer 3, or GPT-3, developed using computer-based processing of huge amounts of publicly available textual data (natural language)^1^, may be coming to a healthcare clinic (or eHealth application) near you. This may sound fantastical, but not too long ago so did a powerful computer so tiny it could fit in the palm of your hand. GPT-3 and other technologies are getting close to passing a Turing Test, an assessment of whether the language such applications generate is indistinguishable from language produced by humans^2,3^. This possibility has generated both excitement and caution^4^, and Microsoft Corporation recently acquired an exclusive license from OpenAI for GPT-3^5^. As with so many technologies and their potential use in eHealth, there are developments and applications that are unrealistic, realistic, and realistic but challenging—and perhaps unwise.

Natural Language Processing (NLP) has a long history in clinical informatics and includes groundbreaking work using computer-based algorithms that compute on text and natural language. There are many clinical applications of NLP including assisting with provider documentation, automated structured chart abstraction, and in machine learning^6^.

Despite the large amount of work in this area, AI that generates text and conversations, such as GPT-3, will not replace a conversation with another human being for the foreseeable future in clinical settings^7^. This means that it cannot interact with patients in lieu of healthcare providers or healthcare support personnel. Interactions with GPT-3 that look (or sound) like interactions with a living, breathing—and empathetic or sympathetic—human being are not^8^. A recent example of this failing was seen in testing the use of GTP-3 for mental health support using a simulated patient; the model supported the patient’s suggestion of suicide^9^. Moreover, language models such as GPT-3 are not grounded in input-diverse datasets (like visual and auditory data)^1^. GPT-3’s self-supervised prediction will, therefore, hit limits based on its pre-training data and cannot dynamically adjust a conversation or interaction for tone or body language.

GPT-3 is an autoregressive language model trained with 175 billion parameters and then tested in “few-shot learning settings” (in which a new language task can be performed after only a few examples). Autoregressive language models predict the next element in a text, usually a word, based on previous natural language texts. Although its developers at OpenAI think it performs well on translation, question answering, and cloze tasks (e.g., a fill-in-the-blank test to demonstrate comprehension of text by providing the missing words in a sentence)^1^, it does not always predict a correct string of words that are believable as a conversation. And once it has started a wrong prediction (ranging from a semantic mistake to using biased language), it does not go back and correct itself but continues to predict each word based on the preceding words. Further, since it is based on real language, human biases are present and, with inadequate priming of the application, may even be amplified and cause serious harm in sensitive contexts, such as healthcare. It is well-known that Internet-trained models reflect the scale of bias seen on the Internet, recently demonstrated by using the Implicit Association Test (IAT) to measure biases in a machine learning model trained on web-based content^10^. Therefore, it is unsurprising that GPT-3 showed associations between gender and certain jobs; often the default was male. Negative sentiments were associated with Black race and positive with Asian race. Islam was more often associated with terrorism-related words than were other religions^1^. Furthermore, according to recent research at the Center on Terrorism, Extremism, and Counterterrorism, GPT-3 is easy to prime for harmful text generation promoting extremism and terrorist activities, including Nazism and QAnon^11^.

It is within this caveat-filled context that evaluation of AI health and healthcare applications that produce natural language should assess their risk, feasibility, and return on investment—including prioritizing improved patient care. Realistic applications of GPT-3 must start in areas of high value, high feasibility, and low risk for all stakeholders, including (at a minimum) patients, clinicians, administrators, and payers. Applications with higher levels of risk or feasibility must be studied extensively and their actual and projected short-, medium-, and long-term impact measured. Realistic but challenging or unwise applications include those that are medium to high feasibility, medium to high risk, and medium to high value.

## Unrealistic applications for GPT-3 applications in healthcare

GPT-3 is not an artificial general intelligence. It will not, and cannot (for now at least), replace a human interaction that requires humanness^12,13^. Although GPT-3 performed well on free-form conversation assessments demonstrating reading comprehension, it performed worst on a dataset meant to mimic the dynamic give-and-take of student-teacher interactions, and it also did not score well on multiple choice questions from middle and high school examinations^1^. This makes sense because it does not “know” anything. One of the major limitations of GPT-3 is that it repeats itself semantically, loses coherence over long conversations, and contradicts itself^1,14^. It would be unrealistic to consider GPT-3 as a stand-in for a healthcare provider or as a proxy in high-stakes interactions, such as a health emergency or an emotionally fraught interaction.

## Realistic and feasible GPT-3 applications in healthcare

There is compelling promise and serious hype in AI applications that generate natural language. Some of that promise is realistic. Routinizing tedious work for providers could productively improve their work satisfaction and reduce time interacting with computer systems, a well-documented concern^15^. AI NLP applications could navigate complex electronic health record (EHR) systems, automate documentation with human review, prepare orders, or automate other routine tasks.

It is, however, capable of more complexity in its text conversations than a chatbot, including more natural-sounding question and answer exchanges^14^. This could personalize the experience of data collection in several non-critical healthcare system encounters, including online chat support for patients or assisting patients with setting up equipment in preparation for a telehealth visit. In fact, its developer, OpenAI, originally intended the software to be used by companies and organizations to improve customer service chatbots or do other similar tasks^16^.

However, each application must also include implementation guidance, including serious guardrails for all healthcare-related interactions. For example, this could mean it would be primed, perhaps using few-shot training alongside imposed limitations, to discuss solely relevant topics—and only after excisions of harmful, prejudicial, or inappropriate vocabulary.

## Realistic but challenging GPT-3 applications in healthcare

Implementation guidance will be even more important in adapting GPT-3 technology for realistic but challenging healthcare applications. For example, using GPT-3 to assist with triaging non-critical patients presenting in the emergency department might seem a good use of the technology, from both a patient experience perspective and a resource allocation perspective. In this example, the focus would be on collecting accurate data from patients in a user-friendly way, thereby improving the patient experience (by making it easier to provide information) and enhancing patient care (by freeing up clinicians to spend more time in meaningful clinical encounters rather than routine data collection).

FDA approval would likely be required in this type of application in the United States and any evaluation must consider a broadly diverse population of patients. For instance, stakeholders, including developers and implementers, will need to be mindful of allocational and representational harms^17,18^, particularly when a virtual agent acts as a gatekeeper^19^—in which case the patient-user has no other option than to interact first with the virtual agent. In the triage example, the allocational harm occurs when those who are more able to successfully interact with the GPT-3 text intake process or forms are more likely to be triaged accurately. Implementation should include another means of triaging those patients who cannot, or do not wish to, use the conversational agent, which may also be too linguistically homogenous to offer culturally mindful language use. Furthermore, alternatives should be readily apparent and easy to access. Although this may seem to duplicate work, it is a necessary step in ensuring that harms are reduced and, as the GPT-3-driven interaction improves, redundant effort should be needed less often. An appropriate staff member would also need to review all triage forms completed using the GPT-3 application; it will be important to maintain a “human in the loop”. A representational harm in this triage example might be when the GPT-3 intake is only available in one language. In such a case, one could explore GPT-3’s expanded languages and translation functions: though the intake interaction could be in Spanish, form material could then be translated into the language spoken by the triage staff. There are real possibilities here, if done well, for language and related healthcare access barriers to be reduced. However, without careful implementation, including the step-wise process and oversight we describe, this triage application would be unwise with the potential to cause both immediate harm to individual patients and broader harm to patient groups, exacerbating healthcare disparities.

## Making sure any realistic applications are equitable

AI software that generates natural language could be viewed as just another vendor-provided information technology tool, but it should not be. The role of human-computer interactions in informing the design of these conversational spaces, whether in an online chat or at an emergency department patient registration kiosk, will be critical to ensure not just that accurate and relevant data are collected, but also that the experience is what diverse users expect, want, and value. A broad range of stakeholders should be involved from the earliest stages of development (or tailoring) through deployment and evaluation. Stakeholders should be selected who can represent as comprehensive a view as possible on both the harms and benefits of proposed uses of GPT-3 in eHealth applications.

Transparency will be key to the appropriate use of GPT-3 types of technology. Human beings must be informed that the interaction is with a computer-based text generator. Doing so would address concerns that humans tend to anthropomorphize technology applications with human traits, assuming humanness and ascribing empathic emotional responses when there are none^20,21^. Some applications are highly feasible and seem low-risk but might harbor hidden hazards. For example, an innocuous natural language clinic appointment scheduler could not, with existing technology, detect a change of tone or social cues of nervousness a patient expresses and that might signal more urgent clinical needs.

Transparency is also critical for datasets and to disclose the limitations in language training activities. A GPT-3 application will need to be given conversation endpoints so that it leads the prompts rather than having the patient control the focus of the interaction; for form-completion tasks, it will also need additional guidance to determine whether the information a patient shares actually addresses the question posed. IT support personnel, or those in similar roles, will need to learn how to shape the prompts that will deliver the most relevant answers or results from a given interaction. For GPT-3 priming using few-shot learning, a commitment to transparency would require publishing any customized parameters. In high-risk applications in healthcare, including any patient-facing tools, such sharing must be mandatory.

We should have cautious optimism for the potential applications of sophisticated natural language processing applications to improve patient care. Additional concerns from our triage example include many implementation issues, including the ways AI software would interface with clinical and healthcare support workflows (a known concern for other AI applications^22,23^), how the data will be analyzed in real-time on the backend to successfully triage patients in a queue that prioritizes more concerning symptoms, and the degree and type of liability assigned the health system or provider. The future is coming. Rather than fear it, we should prepare for it—and prepare to benefit humanity using these applications. But for now, Dr. GPT-3 is not coming to a clinic near you anytime soon.
